# Ammonia nitrogen stress damages the intestinal mucosal barrier of yellow catfish (*Pelteobagrus fulvidraco*) and induces intestinal inflammation

**DOI:** 10.3389/fphys.2023.1279051

**Published:** 2023-09-19

**Authors:** Senyue Liu, Lin Luo, Fengyuan Zuo, Xiaoli Huang, Liang Zhong, Sha Liu, Yi Geng, Yangping Ou, Defang Chen, Wenlong Cai, Yongqiang Deng

**Affiliations:** ^1^ Fisheries Research Institute, Sichuan Academy of Agricultural Sciences, Chengdu, Sichuan, China; ^2^ Department of Aquaculture, College of Animal Science & Technology, Sichuan Agricultural University, Chengdu, Sichuan, China; ^3^ State Key Lab of Marine Pollution, Department of Infectious Diseases and Public Health, Jockey Club College of Veterinary Medicine and Life Sciences, City University of Hong Kong, Hong Kong, Hong Kong SAR, China; ^4^ Department of Basic Veterinary, College of Veterinary Medicine, Sichuan Agricultural University, Chengdu, Sichuan, China

**Keywords:** ammonia nitrogen, mucosal immune, mucosal barrier damage, intestinal inflammation, *Pelteobagrus fulvidraco*

## Abstract

Nitrogen from ammonia is one of the most common pollutants toxics to aquatic species in aquatic environment. The intestinal mucosa is one of the key mucosal defenses of aquatic species, and the accumulation of ammonia nitrogen in water environment will cause irreversible damage to intestinal function. In this study, histology, immunohistochemistry, ultrastructural pathology, enzyme activity analysis and qRT-PCR were performed to reveal the toxic effect of ammonia nitrogen stress on the intestine of *Pelteobagrus fulvidraco*. According to histological findings, ammonia nitrogen stress caused structural damage to the intestine and reduced the number of mucous cells. Enzyme activity analysis revealed that the activity of bactericidal substances (Lysozyme, alkaline phosphatase, and ACP) had decreased. The ultrastructure revealed sparse and shortened microvilli as well as badly degraded tight junctions. Immunohistochemistry for ZO-1 demonstrated an impaired intestinal mucosal barrier. Furthermore, qRT-PCR revealed that tight junction related genes (*ZO-1, Occludin, Claudin-1*) were downregulated, while the pore-forming protein *Claudin-2* was upregulated. Furthermore, as ammonia nitrogen concentration grew, so did the positive signal of Zap-70 (T/NK cell) and the expression of inflammation-related genes (*TNF*, *IL-1β*, *IL-8, IL-10)*. In light of the above findings, we conclude that ammonia nitrogen stress damages intestinal mucosal barrier of *Pelteobagrus fulvidraco* and induces intestinal inflammation.

## Graphical Abstract

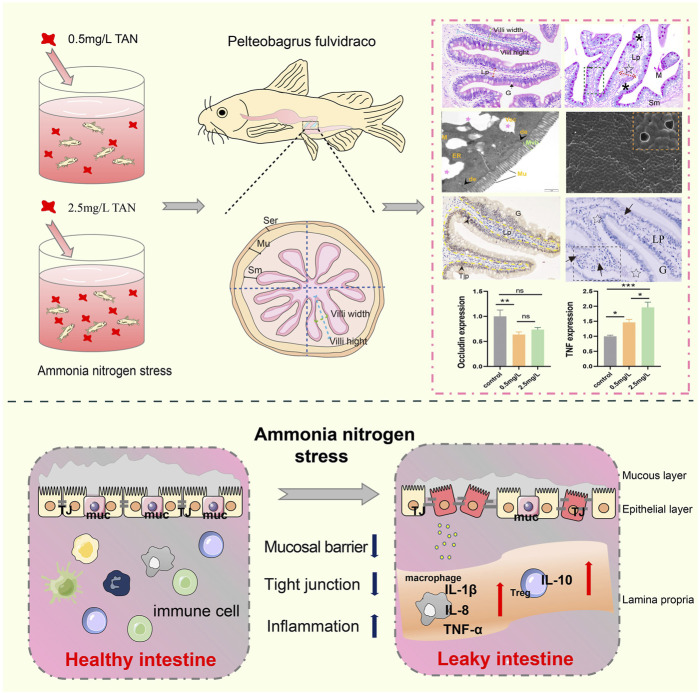

## 1 Introduction

Nitrogen sources derived from ammonia have been utilized to assess water quality in the aquaculture industry, and it is regarded as a long-term contaminant in the aquatic environment (Zhang et al., 2021). In aquaculture water environment, ammonia nitrogen exists in the form of non-ionic ammonia (NH_3_) and ionic ammonia (NH_4_^+^) which converts to each other and maintains dynamic balance under specified pH, temperature, and salinity parameters (Kleinhenz et al., 2018; Hongxing et al., 2021). However, due to aquatic creatures’ exceptional sensitivity to ammonia nitrogen, excessive quantities of ammonia nitrogen can be hazardous to them, with NH3 being the primary source of ammonia toxicity (Kathyayani et al., 2019; Zhang et al., 2020). At specific water temperatures and pH levels, NH_3_ diffuses through biological cell membranes more easily than NH_4_^+^, leading to metabolic alterations, oxidative stress, inflammation and disease (Lu et al., 2022).

Intestine, one of the body’s first lines of defense, is the main route for many aquatic animals to absorb environmental pollutants (Pustiglione Marinsek et al., 2018). The variety of intestinal microbiota, tissue structure, and physiological function will alter in response to changes in the aqueous environment, and can be utilized as a reflection of environmental pollution. Therefore, intestine is a vital organ for assessing water environmental pollutants (Gonçalves et al., 2020; Zhang et al., 2020). Intestinal mucosal is critical in maintaining homeostasis. In general, intestinal mucosal barrier system can be divided into biological barrier, chemical barrier and mechanical barrier. Biological barrier refers to the parasitic bacteria residing in the intestine with colonizing resistance to foreign strains (Bischoff et al., 2014). Chemical barrier includes mucins, defencins, lysozyme, alkaline phosphatase and other bacteriostatic substances (Ghosh et al., 2021), which are not only beneficial to prevent microbial invasion, but also play a coordinating role in the immune defense process (Wu et al., 2019). The mechanical barrier is composed primarily of intestinal epidermal cells and intercellular junction components including attachment junctions (AJ), tight junctions (TJ), and desmosome. They play vital roles in maintaining intestinal epidermal permeability and preventing intestinal lumen substances from entering the intestine (Camilleri et al., 2012). Numerous studies have shown that ammonia nitrogen accumulation in aquatic environments can irreversibly damage the normal structure and barrier function of intestine (Wood et al., 2019; Qian et al., 2021; Wang et al., 2021).

Yellow catfish (*Pelteobagrus fulvidraco*) is an essential component of China’s freshwater ecosystem and a significant economic species, with a total output of 560,000 t in 2020 (Administration, 2021). As a representative of benthic animals in aquatic ecosystems, yellow catfish can be utilized to evaluate the bioaccumulation rate of contaminants in water, providing a suitable model for environmental monitoring (Chen et al., 2012; Kim et al., 2012). However, with the rapid development of high-density intensive farming, ammonia nitrogen accumulation has become a universal problem for ecology and aquaculture, leading to environmental pollution (Li et al., 2020). For example, diseases increased during the breeding of yellow catfish due to increasing ammonia stress, resulting in a mortality rate of over 70% and massive economic losses (Chen et al., 2016). Although many studies on the toxic effects of ammonia nitrogen on aquatic animals have been undertaken, few studies have focused on the impact of ammonia stress on intestinal histology in fish. In addition, how ammonia nitrogen affects the intestinal mucosal barrier of yellow catfish and its related mechanisms remain unclear. Therefore, it is particularly important to elucidate the toxic effect of ammonia nitrogen on the intestine of yellow catfish*,* which can not only help to assess the potential risks of ammonia nitrogen stress on aquatic animals, but also provide insights for management strategies and intervention targets for ammonia nitrogen-induced stress.

## 2 Materials and methods

### 2.1 Experimental fish

Yellow catfish (53.67 ± 4.90g) used in this study were obtained from a fish farm in Chengdu (Sichuan province, China), with no superficial injuries. Five fish were randomly selected for examination, no bacteria or parasites were found. These fish were kept in a circular tank (600 L water volume) with constant aeration for 1 week at 23°C–24°C. Floating commercial feed was provided satiably twice a day (8 a.m. and 6 p.m.), with one third of the water was changed daily. The approximate composition of commercial feed is as follows: 40% crude protein, 5% crude fat, 8.1% ± 0.3% crude fibr and 16% crude ash. Feeding was ceased 1 day before ammonia nitrogen exposure.

### 2.2 Ammonia nitrogen exposure experiment and sample collection

Fish were equally divided into three groups: control group (total ammonia nitrogen (TA-N) 0 mg/L), 0.5 mg/L group (TA-N 0.5 mg/L, common stressful and toxic concentration exceeding the standard in modern fisheries), and 2.5 mg/L group (TA-N 2.5 mg/L, 10% 96-h LC_50_). They were kept in three circular tanks, and were continuously aerated. Then, based on previous research (Liu, 2022; Zhong, 2022), a 28-day stress experiment was conducted. Briefly, the TA-N concentration in water was determined using the Nessler reagent-colorimetry method (SI Appendix) (Kołacińska and Koncki, 2014). By adding 10 mg/L ammonium chloride solution as needed, the expected ammonia nitrogen concentration was achieved. The fish were fed with commercial feed in a satiated manner. To reduce the impact of exogenous nitrogen, the unconsumed feed was removed after half an hour of feeding. To reduce the impact of nitrogen in the excrement, water was changed in a 1/3 ratio at 9:00 a.m. every day to remove excrement from the water. To ensure ammonia nitrogen concentration stability, water TA-N concentration was measured twice a day (9 a.m. and 21 p.m.), and immediately adjusted to the specified experimental level. During the experimental period, the water temperature was 23°C–24°C, the dissolved oxygen was 8.0 - 8.9 mg/L, the pH was 6.5-7.1, and the actual ammonia nitrogen concentration is shown in Supplementary Table S1.

After 28 days of ammonia nitrogen exposure, fish were anesthetized with buffered MS222 (250 mg/L; Aladdin, China), and posterior intestine tissue samples were immediately collected for downstream analysis.

### 2.3 Histology, AB-PAS, and immunohistochemical studies

#### 2.3.1 Hematoxylin-eosin staining

**FIGURE 1:**
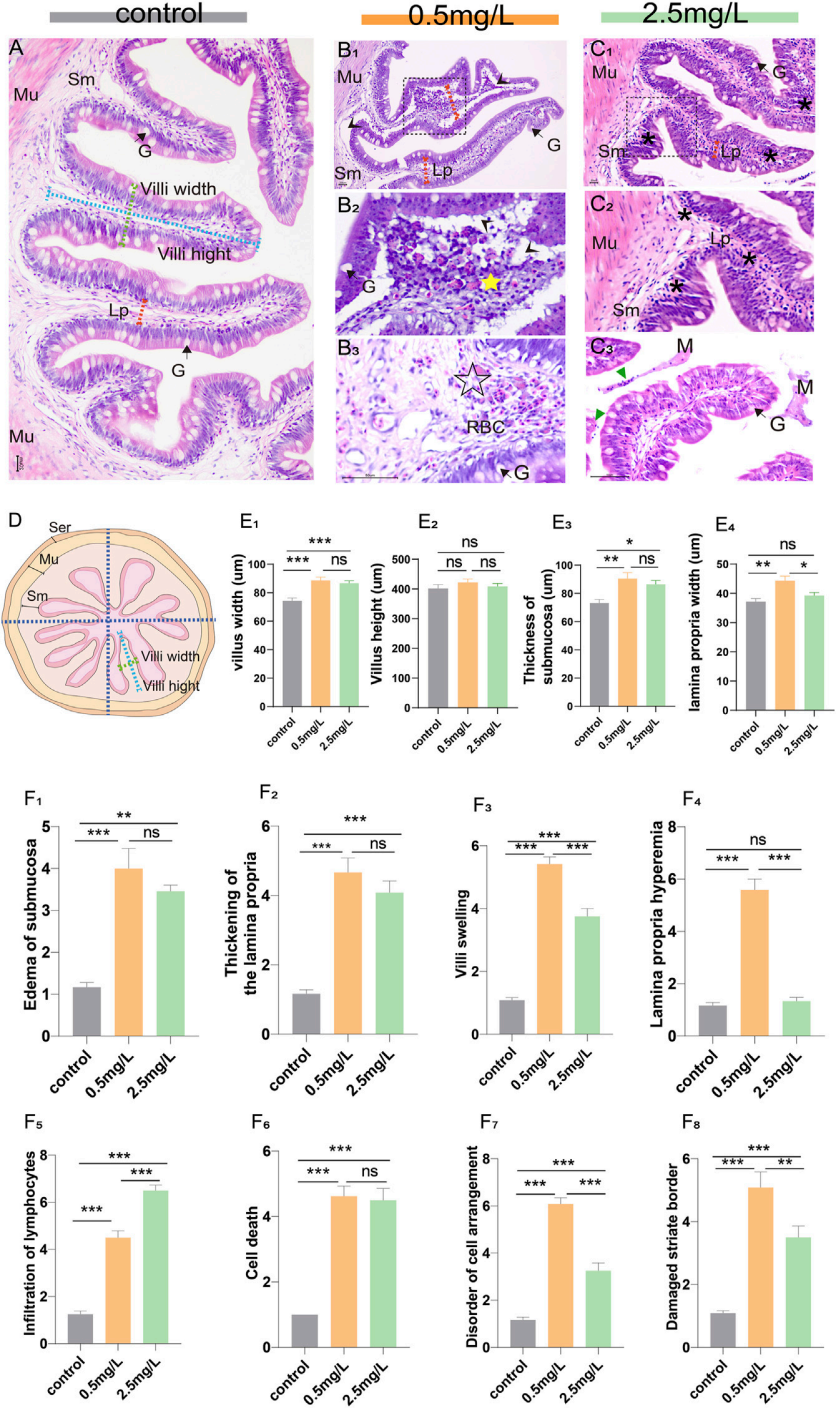
Histopathological observation of intestine of yellow catfish. **(A)** Intestine of the control group, the villi were regular in shape, the dashed blue line, dashed green line, and dashed red arrows represent villi height, villi width, and lamina propria width, respectively. **(B**_**1**_ **-B**_**3**_ **)** Intestine of the 0.5 mg/L group: **(B**_**1**_ **)**, the intestinal villi were severely vacuolated (arrowhead), and the thickness of lamina propria increased (dashed red arrows). **(B**_**2**_ **)**, Local zoom of Fig **(B**_**1**_ **)**, showing disorder of cell arrangement (yellow star), lamina propria edema and vacuolation (arrowhead). **(B**_**3**_ **)**, The lamina propria showed marked hyperemia (☆) with numerous red blood cells. **(C**_**1**_ **-C**_**3**_ **)** Intestine of the 2.5 mg/L group: **(C**_**1**_ **)**, the villi were swollen with marked inflammatory cell infiltration (*). **(C**_**2**_ **)**, Local zoom of Fig **(C**_**1**_ **)**. **(C**_**3**_ **)**, there were necrotic and exfoliated intestinal epithelial cells and inflammatory cells (green triangle) in the intestinal lumen, along with some mucous. **(D)** Schematic of the transverse section of the intestine, which is divided into 4 areas, the dashed blue line, dashed green line, and dashed red arrows represent villi height, villi width, and lamina propria width, respectively. **(E**_**1**_ **–E**_**4**_ **)** Morphological measurements of villi width, villi height, thickness of submucosa, and lamina propria width, respectively. **(F**_**1**_ **–F**_**8**_ **)** The histopathological scores for edema of submucosa, thickening of the lamina propria, villi swelling, lamina propria hemorrhage, infiltration of lymphocytes, cell death, disorder of cell arrangement, and damaged striate border, respectively. Sm, submucosa; Mu, muscularis; G, goblet cell; Lp, lamina propria; M, mucosa; RBC, red blood cell. *, **, ***, and ns representing *p* < 0.05, 0.01, 0.001, and nonsignificant, respectively. (*n* = 6).

Eighteen intestine samples (6 samples per group) were obtained for histopathological analysis. Briefly, the posterior intestines were removed with sterile forceps, immediately prefixed in 4% paraformaldehyde for 24 h, rinsed in running tap water for 24 h, and then routine dehydration and paraffin embedding were performed. Subsequently, the tissues were sliced with a thickness of 5 μm (Lycra, Germany), and stained with classical hematoxylin and eosin (H & E). Photographs were taken under an optical microscope (Nikon Eclipse E200, Japan). Each tissue section was divided into 4 areas (Figure 1D), and a single intestinal villus was randomly selected from each area. ImageJ was used for morphological measurements (including villi height, villi width, submucosal thickness and lamina propria width), and pathological changes of intestinal tissues (including edema of submucosa, thickening of the lamina propria, villi swelling, lamina propria hemorrhage, infiltration of lymphocytes, cell death, disorder of cell arrangement, and damaged striate border) were analyzed by pathological score. The severity of the lesions is indicated by a score (S) ranging from 1 to 7 (Barišić et al., 2018): (1) unaltered; (3) mild; (5) moderate; and (7) severe.

#### 2.3.2 Alcian blue and periodic acid–schiff staining

The above paraffin sections of intestinal samples were dewaxed in xylene, hydrated, and stained for 20 min with Alcian blue solution (pH 2.5). Later, the samples were oxidized in Periodic acid (5 g/L) for 5 min, rinsed with distilled water for 10 min, and immersed in Schiff’s reagent for 20 min under dark conditions before being washed with distilled water. Finally, the samples were stained with Hematoxylin for 2 min before being sealed with neutral gum. Nikon Eclipse E200 (Japan) was used to evaluate tissue slides. Each tissue section was divided into 4 areas (Figure 1D), and a single intestinal villus was randomly selected from each area. The thickness of the epithelial layer and the number of mucus cells of a single villus were measured and recorded.

#### 2.3.3 Immunohistochemical studies

The above paraffin sections were dewaxed to water and then placed in a repair box containing citric acid antigen repair buffer (pH 6.0). Then, the slices were placed in 3% hydrogen peroxide solution for 25 min before being rinsed three times in PBS (pH 7.4) for 5 min each to block endogenous peroxidase. Afterward, the tissues were evenly covered with 3% BSA (Thermo Fisher, United States) and sealed at room temperature for 30min. The primary antibodies Zap-70 Rabbit monoclonal antibody (99f2, CST, Massachusetts, United States) and ZO-1 Rabbit polyclonal antibody (GB111402, Servicebio, China) were added and incubated overnight in a wet box at 4 °C. After washing, tissues were covered with Goat Anti-Rabbit IgG (H&L) Alexa Flour 488secondary antibody (Thermo Fisher, United States), and incubated at room temperature for 50min. Following PBS washing, sections were immersed in diaminobenzidine hydrochloride (DAB) and re-stained with hematoxylin. The sections were observed under Nikon Eclipse E200 (Japan). Each tissue section was divided into 4 areas (Figure 1D), and a single intestinal villus was randomly selected from each area. ImageJ was used to assess mean optical density (IOD SUM/area), and IHC Profiler was used to assess immunohistochemistry score (IHC score) (Varghese et al., 2014). The following are the scoring criteria: (3) strongly positive; (2) positively; (1) moderately positive; and (0) negatively.

### 2.4 Transmission electron microscopy (TEM)

Eighteen intestine samples (6 samples per group) were selected for transmission electron microscopy (TEM) analysis. The samples were fixed in 2.5% glutaraldehyde for 24 h at 4°C before being washed with PBS (pH 7.2). They were then fixed with 1% osmic acid, washed with PBS, dehydrated with continuous acetone, embedded, sliced, and stained with uranium acetate and lead citrate. Micrographs were taken with TEM (Hitachi H-7500, Japan) operating at 80 kV. The length of microvilli and the diameter of mucinous granules were measured and recorded using ImageJ software. The ultrastructural pathological changes of intestinal tissue were evaluated, including necrosis, tight junctions fuzzy, vacuolization, mitochondrial cristae contraction and swelling, endoplasmic reticulum swelling, mitochondrial necrosis, mitochondrial myelinoid lesions, autophagy and apoptosis. The samples were assessed using a scoring system (Zhang et al., 2011) ranging from 1 to 7, depending on the severity of the lesions: (1) unaltered; (3) mild; (5) moderate; and (7) severe.

### 2.5 Scanning electron microscopy (SEM)

SEM analysis was performed on eighteen intestine samples (6 samples per group). The intestines were cut into 5-mm size pieces, washed in 1% S-carboxymethyl-L-cysteine for 30s to remove mucus before being stored in 2.5% glutaraldehyde sodium bicarbonate buffer (0.1 M pH 7.2). Subsequently, the samples were dehydrated in ascending series of ethanol before being dried with liquid CO_2_ in a critical point dryer (HCP-02 Hitachi). The samples were scanned at 20 kV with a FEI Inspect S50 SEM (FEI, United States). The density of microvilli on the surface of intestinal cells (0.25 μm^2^ region) was assessed using SEM images (magnification 30,000).

### 2.6 Biochemical analysis

Eighteen intestine samples (6 samples per group) were selected for biochemical analysis. After homogenizing the samples with ice-cold physiological saline (1:19, wt/vol), they were centrifuged at 8,000 rpm for 15 min. The Lysozyme (LZM), alkaline phosphatase (AKP) and acid phosphatase (ACP) activities of tissue supernatant were examined with lysozyme assay kit (A050-1-1), alkaline phosphatase assay kit (A059-2-2) and acid phosphatase assay kit (A060-2-1) respectively, following the manufacturer’s instructions (Jian Cheng Bioengineering Institute, Nanjing, China).

### 2.7 Total RNA extraction and cDNA synthesis

Nine intestine samples (3 samples per group) were randomly selected for extraction of RNAs using TRIzol reagent (Invitrogen, United States) according to the manufacturer’s instructions. Subsequently, the concentration and purit of RNA as well as the RIN value were determined using a Nanodrop 2000 spectrophotometer (Thermo Scientific, United States), a 1.2% (w/v) agarose gel electrophoresis, and an Agilent 2,100, respectively. To remove gDNA, an equal amount of total RNA (1g) was incubated with RNase-Free ddH20 and gDNase Mix. Using the Superscript first strand synthesis system (Abm, Canada), reverse transcription was performed in a 20 μL reaction volume containing 10 μL of the RNA template, 4 μL of the 5 x RO-Easy^TM^ Mix and 6 μL of RNase-Free ddH_2_0.

### 2.8 Quantitative real-time PCR (qRT-PCR) analysis

Quantitative real-time PCR (qRT-PCR) was performed to analyze the expression of immune-related genes (*IL-1β, IL-10, IL-8, TNF-α*) and tight junctions-related genes (*ZO-1, Occludin, Claudin-1, Claudin-2*). The *β-actin* gene of yellow catfish was employed as an internal reference to normalize gene expression levels (Zhou et al., 2021). qRT-PCR was performed in a total volume of 10 μL containing 5 μL of TB Green™ Premix Ex Taq™ II, 0.2 μL of Rox, 1 μL of cDNA, 0.8 μL of each primer (specific primers outlined in Supplementary Table S2) and 2 μL of double distilled water. The reaction conditions used were as follows: 95°C for 3 min, followed by 39 cycles of 95°C for 10 s, 57°C for 20 s and 72°C for 20 s, with the dissolution curve increasing from 0.5°C to 95°C every 5 s, and the gene expression was estimated by the 2^−ΔΔCT^ method (Livak and Schmittgen, 2001).

### 2.9 Statistical analyses

In this study, all data were presented as mean ± SD (standard deviation). SPSS 27.0 software (IBM Corp., Chicago, United States) was used to assess the statistical differences. GraphPad Prism (United States) and Adobe Illustrator (United States) software were used to create the charts. After normality test, the one-way ANOVA analysis was used to evaluate the significant difference. (*, **, ***, and ns representing *p* < 0.05, 0.01, 0.001, and nonsignificant, respectively.)

## 3 Results

### 3.1 Histopathological observation

The structure of the intestinal wall can be divided into four layers based on histological observation: the mucosal layer, the submucosal layer, the muscular layer, and the serosal layer. In the control group (Figure 1A), the villi were nicely organized and the epithelial cells were intact. However, the intestinal lesions in the two-ammonia nitrogen-exposed groups were distinct. In the 0.5 mg/L group (Figure 1B), there was intestinal villus edema (Figure 1B_2_, E_1_), marked submucosal dilatation (Figure 1E_3_), lamina propria thickening (Figure 1B_1_, E_4_) and severe hyperemia (Figure 1B_3_). Interestingly, in the 2.5 mg/L group, the cell composition was relatively uniform, and the most noticeable histopathological change was inflammatory cell infiltration (Figure 1C). In brief, exposure to different concentrations of ammonia nitrogen resulted in disparate types of lesions in the intestine of yellow catfish (Figures 1F; Supplementary Figure S1). Low concentrations (0.5 mg/L) mainly caused reversible changes such as lamina propria hemorrhage, vacuolation and edema, while high concentrations (2.5 mg/L) mainly led to inflammation.

### 3.2 Ammonia nitrogen exposure leads to defects in the intestinal mucosal chemical barrier

**FIGURE 2:**
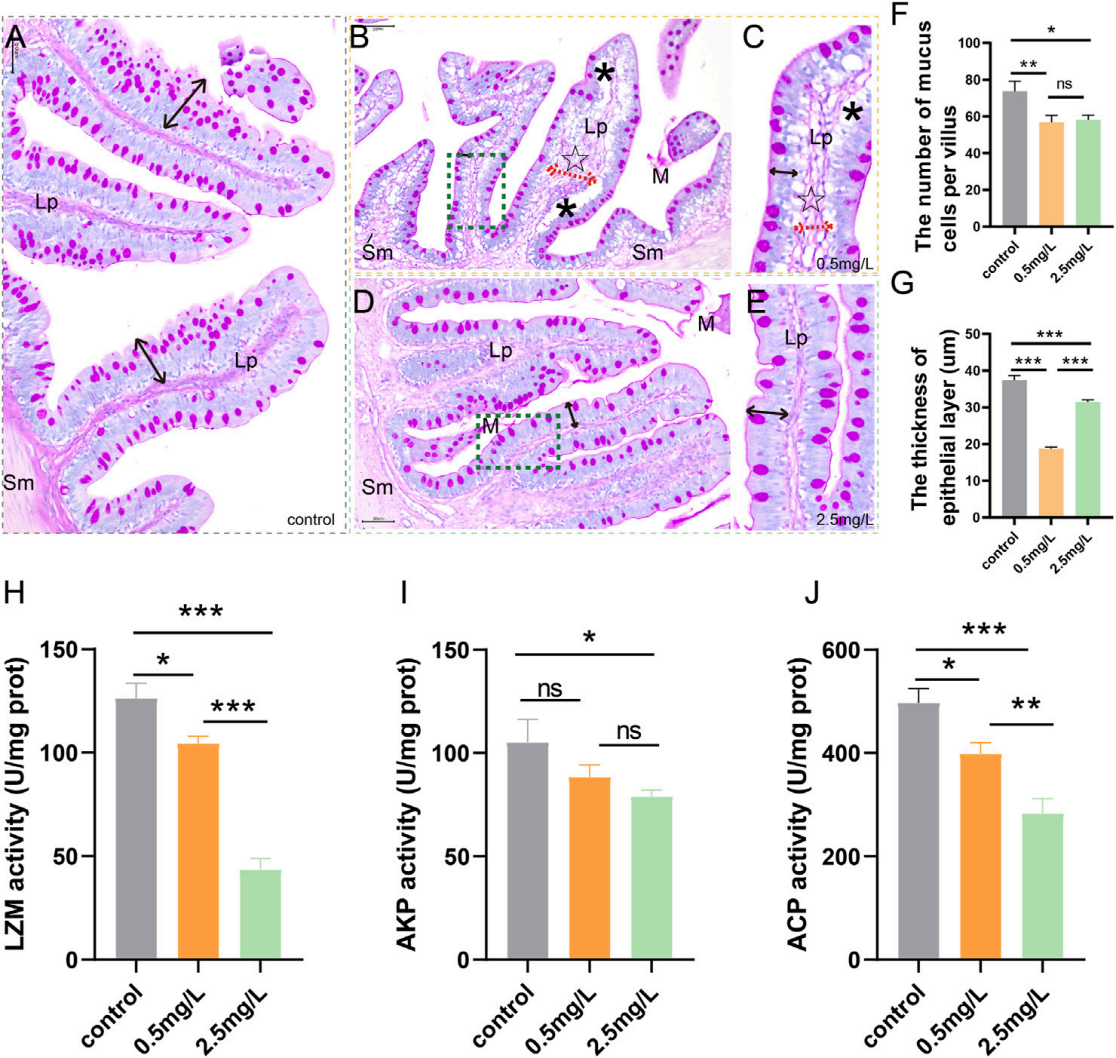
AB-PAS staining results and activity of bacteriostatic substances of intestine after ammonia nitrogen exposure **(A)** The control group showed abundant mucus cells and thick epithelial layer (black double-headed arrow). **(B)** The intestine of 0.5 mg/L group, showed vacuolation (*), sparse mucous cells, and thickened lamina propria (dashed red arrows, ☆). **(C)** Local zoom of Fig B, significantly reduced epithelial layer thickness (black double-headed arrow). **(D)** The intestine of 2.5 mg/L group, showed decreased mucous cells, and moderately decreased epithelial layer thickness (black double-headed arrow). **(E)** Local zoom of Fig **(D) (F)** Mucous cell count in intestine tissue. **(G)** Statistical analysis of epithelial layer thickness. **(H–J)** Activities of LZM, AKP and ACP, respectively. Sm, submucosa; Lp, lamina propria; M, mucosa.*, **, ***, and ns representing *p* < 0.05, 0.01, 0.001, and nonsignificant, respectively. (*n* = 6).

To investigate whether ammonia nitrogen exposure may damage the chemical barrier of intestinal mucosa, AB-PAS staining and enzyme activity analysis were performed. According to the AB-PAS staining results, in the control group (Figure 2A), the epithelial layer was relatively thick and the mucus cells were evenly distributed on the mucosal surface. In the low concentration (0.5 mg/L) group (Figures 2B, C), mucous cells were sparsely distributed and decreased in number, and the thickness of epithelial layer was markedly reduced. Compared with the control group, the number of mucous cells in the high ammonia nitrogen concentration (2.5 mg/L) group was also decreased (Figures 2D, E), and the thickness of epithelial layer was moderately reduced. In brief, after ammonia nitrogen exposure, the number of mucus cells (Figure 2F) and the thickness of epithelial layer (Figure 2G) decreased, especially in low concentration group.

According to the results of enzyme activity analysis, the activities of LZM (Figure 2H), AKP (Figure 2I) and ACP (Figure 2J) in the intestine tissues decreased gradually with increasing ammonia nitrogen concentration, indicating that ammonia nitrogen significantly inhibited the activity of intestinal bacteriostatic substances and damaged the chemical barrier of mucosa.

### 3.3 Ammonia nitrogen exposure leads to defects in the intestinal mucosal physical barrier

**FIGURE 3:**
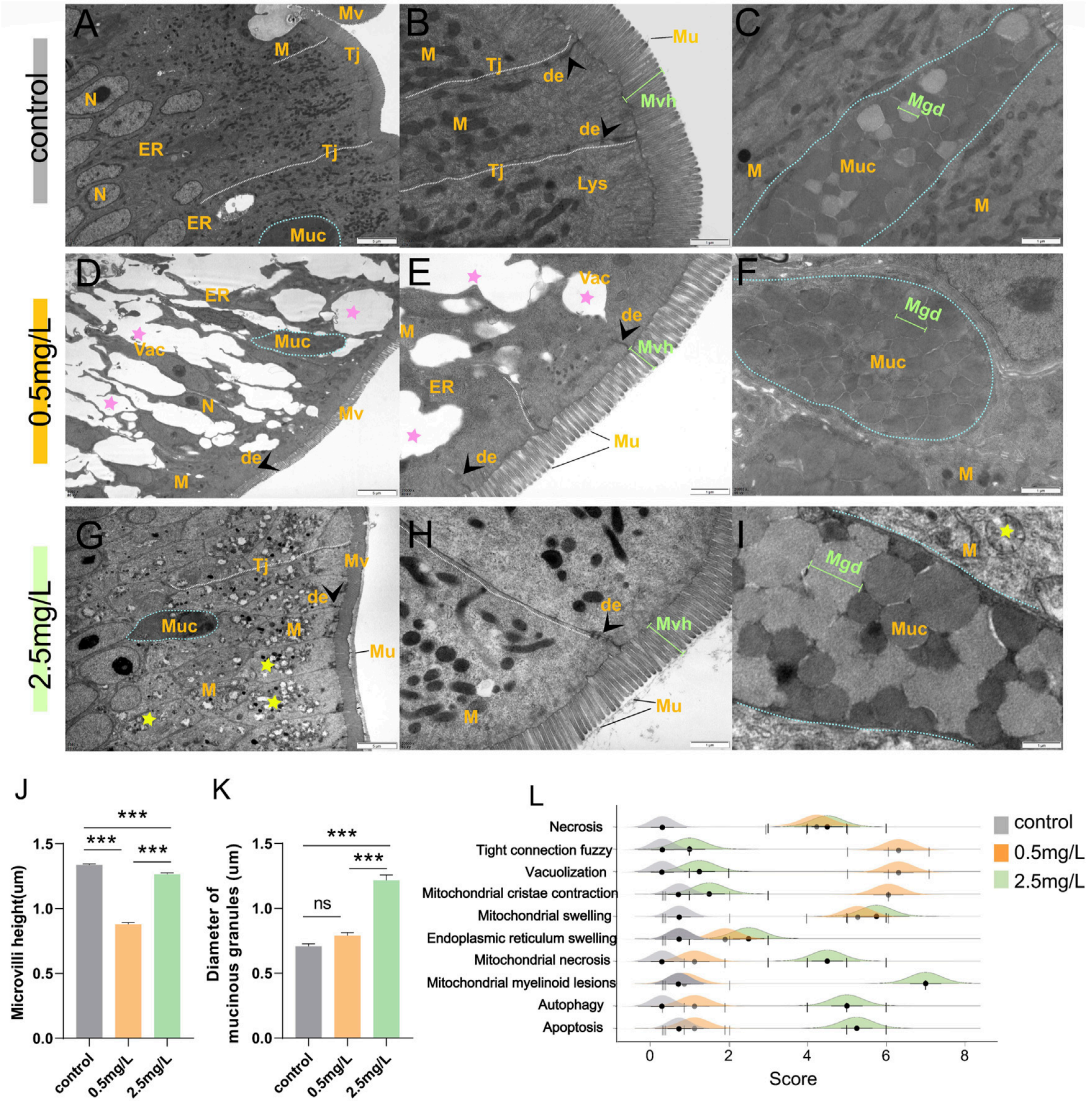
Transmission electron microscope observation of intestine after ammonia nitrogen exposure. **(A–C)** TEM results of intestine in control group: **(A)** Tight junctions were intact (white dotted line), organelles were abundant, and cell morphology was regular; **(B)** Tight junctions were complete (white dashed line) with visible desmosomes (arrowhead), microvilli were dense and long in height; **(C)** Mucus cells (blue dotted line) contained abundant mucus particles. **(D–F)** TEM results of intestine in 0.5 mg/L group: **(D)** The tight junctions were blurred and showing obvious vacuolation (pink star), and few organelles; **(E)** Some tight junctions (white dashed line) disappeared; the microvilli were sparse with significantly reduced height; **(F)** Mucus cells (blue dotted line) contained dark mucus granules. **(G–I)** TEM results of intestine in 2.5 mg/L group: **(G)** The tight junctions (white dotted line) were relatively intact, but a large number of mitochondria showed internal vacuolation, membrane damage, and myelinoid lesions (yellow star); **(H)** The microvilli were slightly sparse and shortened; **(I)** Mucus cells (blue dotted line) contained dark and pale mucus granules. **(J)** Statistical analysis of microvilli height. **(K)** Statistical analysis the diameter of mucinous granules. **(L)** Intestinal ultrastructural pathological scores of the three groups. N, nucleus; M, mitochondria; ER, endoplasmic reticulum; Tj, tight junctions; Mv, microvilli; Muc, mucus cells; de, desmosomes; Lys, lysosomes; Mvh, microvilli height; Mgd, mucinous granules diameter.*, **, ***, and ns representing *p* < 0.05, 0.01, 0.001, and nonsignificant, respectively. (*n* = 6).

To investigate the effect of ammonia nitrogen exposure on ultrastructural structure and physical barrier, transmission electron microscopy (TEM) and scanning electron microscopy (SEM) were performed. According to the TEM results, in the control group (Figures 3A–C), the free surface of intestinal mucosal epithelium was densely arranged with tidy microvilli (Figure 3A). At the free end of epithelial cells, there were abundant cell-cell junction complexes, including tight junctions and desmosomes, as well as a large number of mitochondria with extremely high electron density (Figure 3B). In the 0.5 mg/L group (Figures 3D–F), the intestinal epithelium was severely vacuolated (Figure 3D), and the tight junctions were blurred (Supplementary Figure S1C) or even broken (Supplementary Figure S1D). In addition, the microvilli were sparsely arranged with significantly shortened height (Figure 3E). Interestingly, in the 2.5 mg/L group (Figures 3G–I), the tight junctions of the intestinal epithelium were relatively complete, the microvilli were arranged neatly, and the height was slightly shortened (Figure 3H). The main characteristics were diffused mitochondria vacuolation (Figure 3G) and significant increase in the diameter of mucous particles (Figures 3I, K). In addition, a variety of pathological changes (Figures 3L; Supplementary Figure S2) including necrosis, mitochondrial cristae contraction, mitochondrial swelling, endoplasmic reticulum swelling, mitochondrial necrosis, autophagy and apoptosis were also discovered. In brief, exposure to ammonia nitrogen severely damaged intestinal epithelium, resulting in shortened microvilli height (Figure 3J) and enlarged mucous particle diameter (Figure 3K).

**FIGURE 4:**
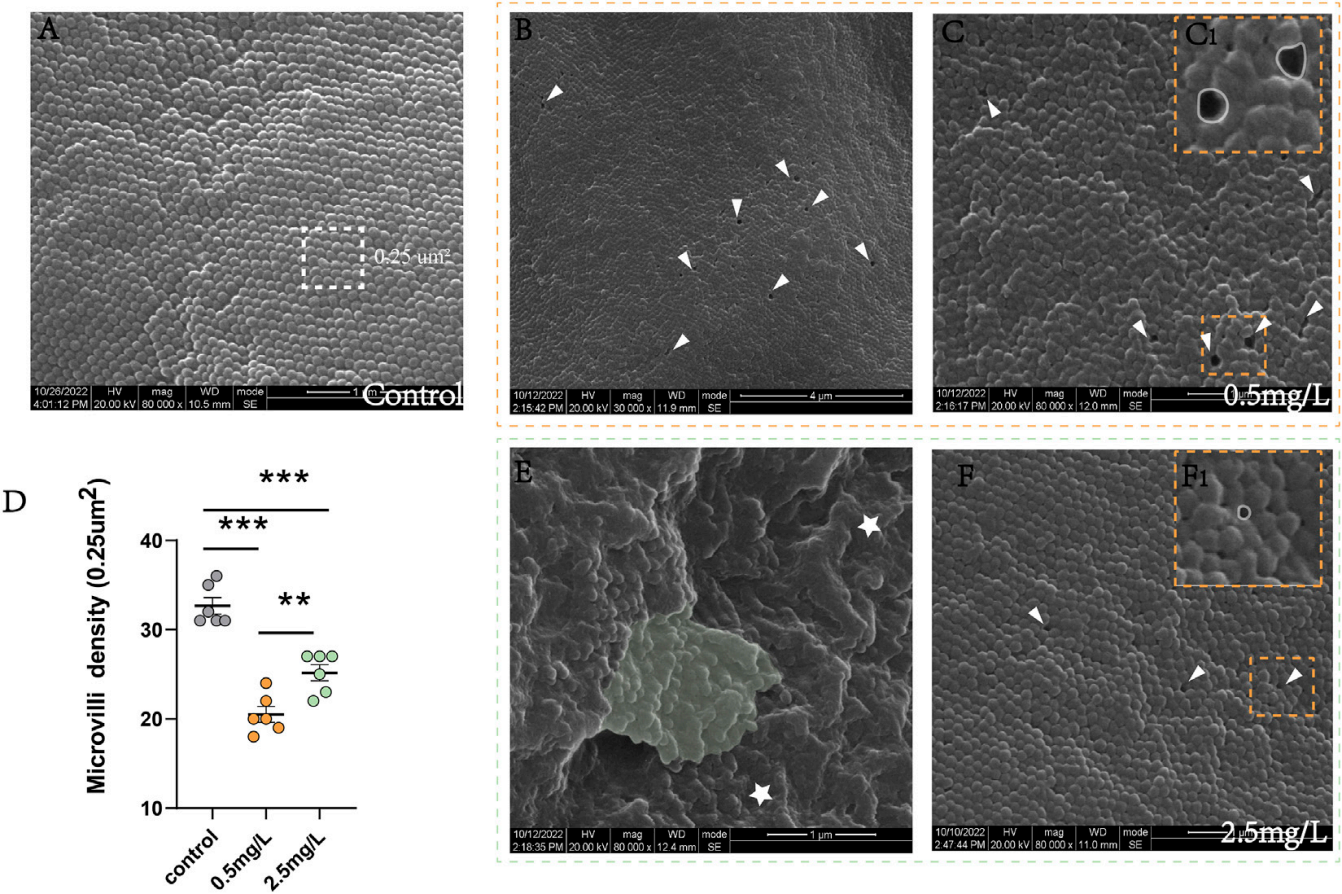
Scanning electron microscope observation of intestine after ammonia nitrogen exposure **(A)** The microvilli in control group were compact and neatly arranged. **(B–C)** The microvilli in 0.5 mg/L group: **(B)** The microvilli showed extensive cavities (white triangle); **(C)** The microvilli were slightly swollen with cavities (white triangle); (C_1_) Local zoom of Fig C, the diameter of the cavity was long (white circle). **(D)** Statistical analysis of microvilli density (0.25 um^2^). **(E–F)** The microvilli in 2.5 mg/L group: **(E)** The microvilli were obviously swollen (star) and adhered to each other (green background); **(F)** Occasionally small cavities (white triangle) were seen between the microvilli; (F_1_) Local zoom of Fig F, the diameter of the cavity diameter was shorter (white circle). *, **, ***, and ns representing *p* < 0.05, 0.01, 0.001, and nonsignificant, respectively. (*n* = 6).

The results of SEM showed that the intestinal microvilli of the control group (Figure 4A) were neat and compact, while samples in the ammonia nitrogen-exposed group showed obvious pathological changes and severe physical damage to the epithelial barrier. Under low concentration of ammonia stress (Figures 4B, C), the microvilli density decreased sharply (Figure 4D), and considerable number of cavities could be visible (Figure 4C). Under high concentration stress (Figures 4E, F), the microvilli swelled and attached to each other (Figure 4E), with occasional cavities (Figure 4F). And the microvilli density was significantly lower than that of control group, but higher than that of 0.5 mg/L ammonia-exposed group (Figure 4D).

### 3.4 Ammonia nitrogen exposure breaks the tight junctions between intestinal mucosal epithelial cells

**FIGURE 5:**
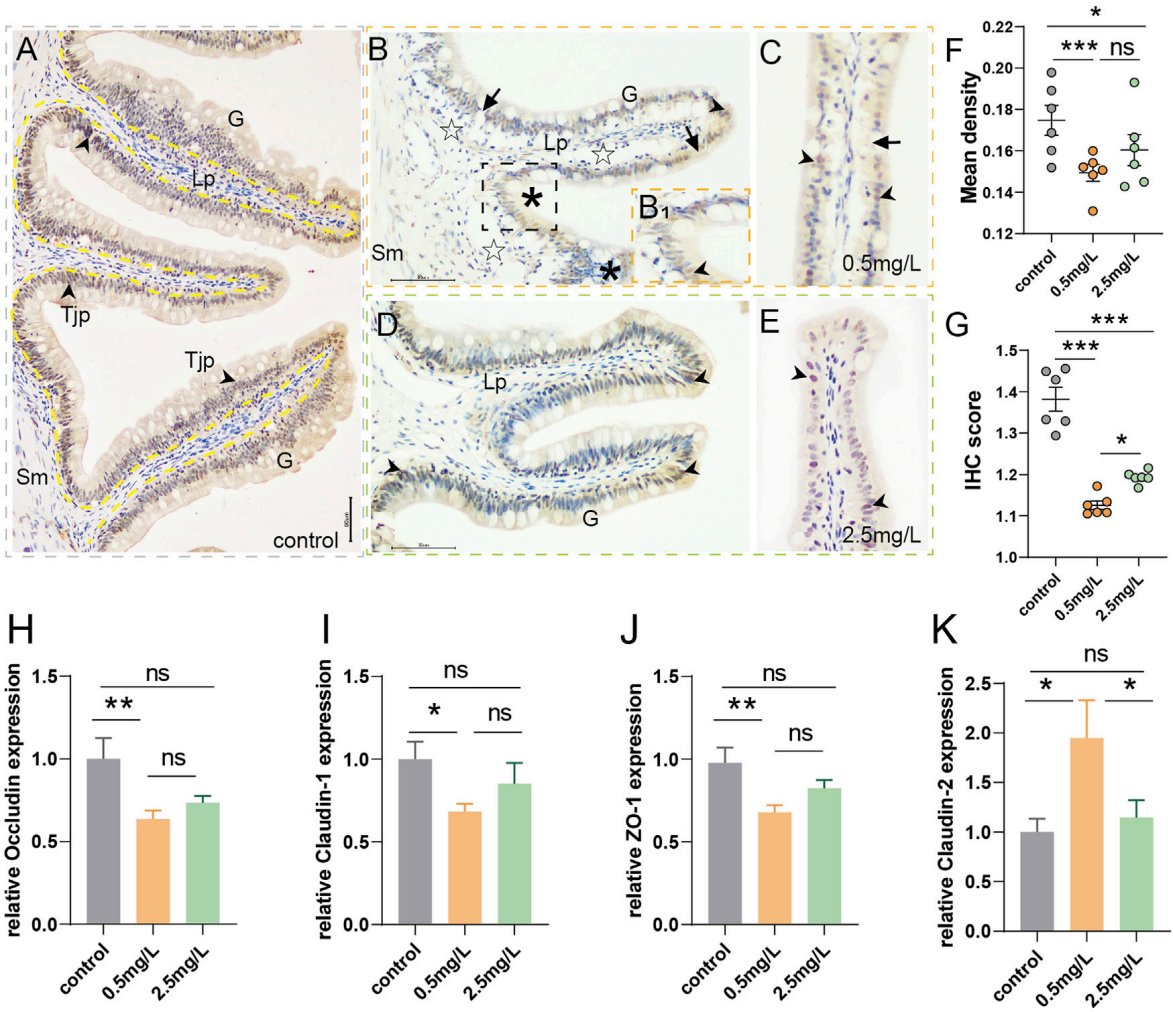
Immunohistochemical observation of ZO-1 (brown) and tight junction-related gene expression **(A)** Immunohistochemical analysis of control group, showed abundant ZO-1^+^ cells (arrowhead) with intact and thick mucosal barrier (yellow dotted line, black arrow). **(B, C)** Immunohistochemical analysis of 0.5 mg/L group: **(B)** Showed obvious vacuolation (☆), a small number of ZO-1^+^ cells (arrowhead), and gaps in mucosal barrier (arrows); B_1_, Local zoom of Fig B, some tight junction proteins migrated up to the surface of goblet cells (*); **(C)** Showed incomplete mucosal barrier (arrows) and sparse tight junction proteins (arrowhead). **(D, E)** Immunohistochemical analysis of 2.5 mg/L group: **(D)** Showed moderate number of ZO-1^+^ cells (arrowhead), **(E)** Showed relatively complete mucosal barrier (arrows). **(F)** Statistical analysis of mean optical density. **(G)** Statistical analysis of IHC score. (*n* = 6) **(H–K)** *Occludin* expression, *Claudin-1* expression, *ZO-1*expression, *Claudin-2* expression, respectively. (*n* = 3) Sm, submucosa; G, goblet cell; Lp, lamina propria; Tjp, tight junction protein. *, **, ***, and ns representing *p* < 0.05, 0.01, 0.001, and nonsignificant, respectively.

To investigate whether ammonia nitrogen exposure could cause damage to the tight junctions between intestinal mucosal epithelial cells, immunohistochemical analysis and qRT-PCR were conducted. In the control group (Figure 5A), the mucosal barrier was intact and ZO-1 protein was evenly distributed in the intestinal mucosa. However, the two-ammonia nitrogen-exposed groups showed different degrees of ZO-1 signal attenuation. In the low concentration group (Figures 5B,C), the positive signal of ZO-1 decreased sharply, and some mucosal barriers were broken. In the high concentration group (Figures 5D,E), the positive signal of ZO-1 decreased moderately, but the mucosal barrier was relatively intact. Statistical analysis of mean density (Figure 5F) and IHC (immunohistochemical) scores (Figure 5G), showed that ZO-1 signal was strongest in the control group, followed by the 2.5 mg/L group and the 0.5 mg/L group.

In addition, genes associated with the promotion of tight junctions (*Occludin, Claudin-1, ZO-1*) (Figures 5H–J) decreased significantly in the 0.5 mg/L group compared to the control group, and also showed a downward but not significant trend in the 2.5 mg/L group. There was no significant difference between the two-ammonia nitrogen-exposed groups. As for *Claudin-2* (Figure 5K), a pore-forming protein that inhibits tight junctions and promotes cell permeability, showed the opposite trend. These findings corroborated immunohistochemistry findings, indicating that ammonia nitrogen exposure (especially at low concentrations) damaged the chemical barrier of intestinal mucosa, affected the formation of tight junctions between cells, and enhanced the paracellular permeability, which might lead to impairment of the intestinal mucosa’s defensive barrier function and induce inflammation.

### 3.5 Ammonia nitrogen exposure induces severe inflammation

**FIGURE 6:**
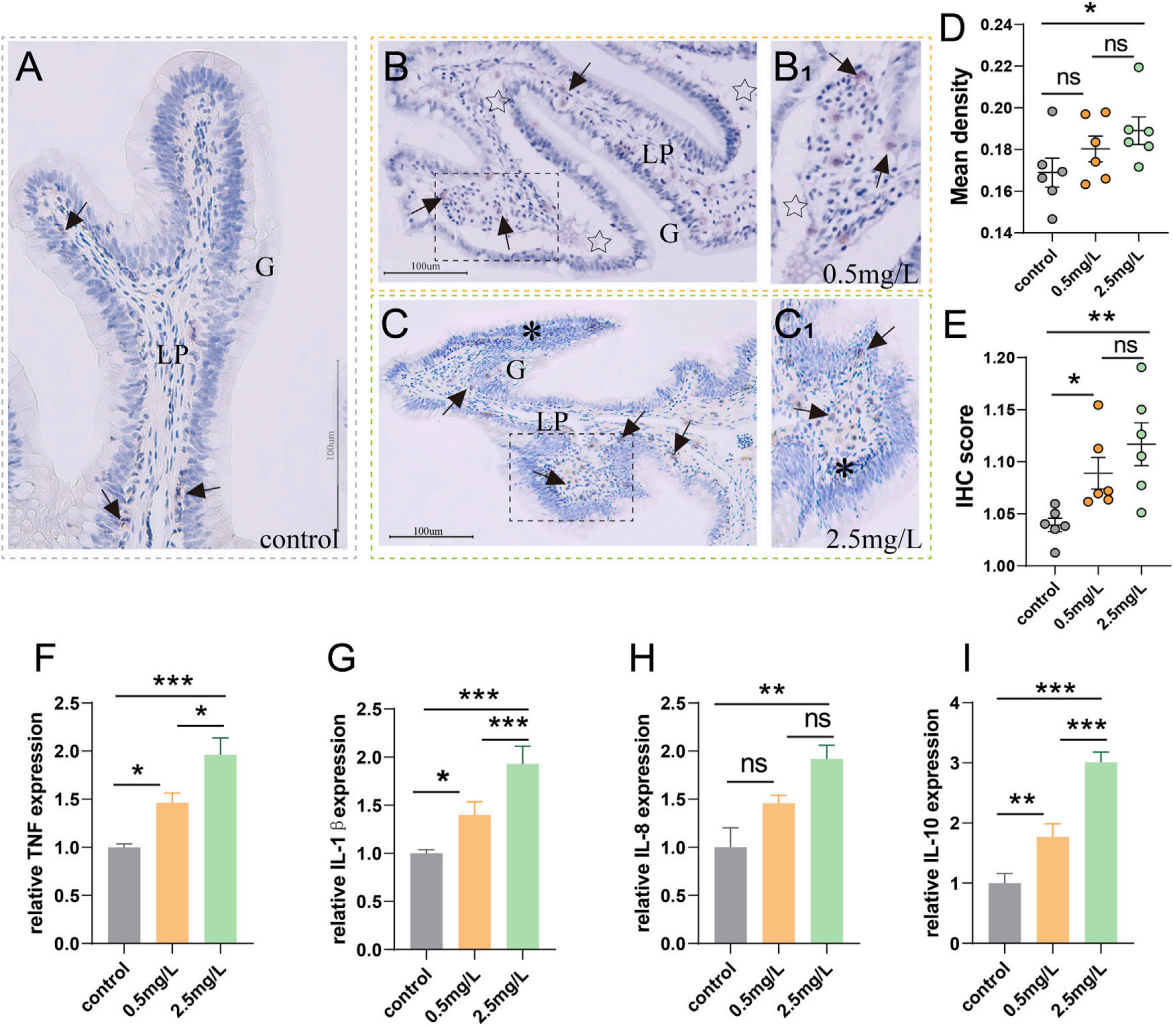
Immunohistochemical observation of Zap-70 (brown) and inflammation-related gene expression **(A)** Immunohistochemical analysis of control group, a few Zap-70^+^ cells (arrows) appeared. **(B)** Immunohistochemical analysis of 0.5 mg/L group, severe tissue vacuolation (star) accompanied by a moderate number of Zap-70^+^ cells (arrows). (B_1_) Local zoom of Fig **(B)**. **(C)** Immunohistochemical analysis of 2.5 mg/L group, showed abundant Zap-70^+^ cells (arrows) and marked lymphocytic infiltration (*). (C_1_) Local zoom of Fig **(C) (D)** Statistical analysis of mean optical density. **(E)** Statistical analysis of IHC score. (*n* = 6) **(F–I)** *TNF-α* expression, *IL-1β* expression, *IL-8* expression, *IL-10* expression, respectively. (*n* = 3) Lp, lamina propria. *, **, ***, and ns representing *p* < 0.05, 0.01, 0.001, and nonsignificant, respectively.

To investigate whether ammonia nitrogen stress could cause intestinal inflammation, immunohistochemical studies and qRT-PCR were conducted. In the three groups (Figures 6A–C), T/NK cells, positive signals of Zap-70 (Lee et al., 2021), were mainly distributed in the gut-associated lymphoid tissue (GALT) of lamina propria. In the control group (Figure 6A), the positive signal was infrequent. In the 0.5 mg/L group (Figure 6B), a moderate number of T/NK cells were scattered in lamina propria, while a large number of T/NK cells gathered in the 2.5 mg/L group (Figure 6C). The statistical results of the mean optical density (Figure 6D) and IHC score (Immunohistochemistry score) (Figure 6E) were comparable with the preceding results, indicating that a large number of lymphocyte aggregation was induced by ammonia nitrogen stress.

According to the results of gene expression, as ammonia nitrogen concentration increased, the expressions of key inflammatory biomarkers (Figures 6F–I) such as *TNF-α*, *IL-1β*, *IL-8* and *IL-10* increased significantly, especially in the 2.5 mg/L group. In light of the aforementioned findings, it is possible that ammonia nitrogen stress triggered severe inflammatory response.

## 4 Discussion

The health of aquatic organisms is positively correlated with water quality and environment (Zimmerli et al., 2007). Since fish are directly exposed to the water, their homeostasis mechanisms are highly dependent on the existing conditions of the surrounding water environment, therefore, even minor changes in water quality can result in a variety of biological responses in fish. Pollution-induced histopathological changes not only reflect the specific effects of pollutants on aquatic organisms, but can also be detected before irreversible effects occur (Wester and Canton, 1991). Therefore, histological methods are considered as sensitive and early warning signals for pollution, and have the advantages of being used to assess potential risks to species survival and environmental protection. Numerous studies have reported that ammonia nitrogen stress has a degenerative effect on fish intestinal tissue. Cao (ShenpingCao et al., 2021) reported that under 50 mg/L ammonia nitrogen stress, the intestinal villi of grass carp swelled substantially and expanded in width. Zhang *et al.* (Zhang et al., 2021) reported that ammonia nitrogen exposure not only caused intestinal inflammation in *Corbicula fluminea*, but also led to changes in the physical structure of intestine, including vacuolation and villi defect. Consistently, similar results were found in this study. After ammonia nitrogen stress, obvious histopathological damage such as vacuolation, intestinal villi swelling, thickening of submucosa and lamina propria, and lamina propria hemorrhage were observed under light microscope. In addition, we also observed significant ultrastructural damage, including autophagy, apoptosis, necrosis, mitochondrial myelination death, and endoplasmic reticulum swelling. These results indicate that ammonia nitrogen stress can seriously damage the intestine of yellow catfish, resulting in severe histopathological changes.

The intestinal epithelium acts as a barrier against the spread of pathogens, toxins, and allergens from the lumen to the mucosal tissues, with mucin and antibacterial compounds serving as the primary components of the intestinal chemical barrier (Peterson and Artis, 2014). Mucus cells secrete mucin, which constitutes the main skeleton structure of the mucus layer and plays an important role in mucosal immunity (Kruatrachue et al., 2003). Therefore, an increase in the number of mucus cells usually leads to increased mucus secretion, which contributes to diluting and detoxifying toxic compounds (Andreozzi et al., 1994). Antibacterial compounds such as ACP, AKP, and LZM are beneficial for local LPS detoxification, and have anti-inflammatory effects (Omonijo et al., 2019). Chen *et al.* (Chen et al., 2011) reported the negative effect of ammonia nitrogen on intestinal chemical barrier of tilapia, and they found the enzyme activities of SOD, LZM, AKP and C3 were significantly reduced after exposure. In this study, after ammonia nitrogen stress, the number of mucus cells and the enzyme activity of antibacterial substances (LZM, AKP, and ACP) were substantially decreased in the intestine of yellow catfish, indicating the degradation of intestinal mucosal chemical barrier.

Tight junctions, including claudin family proteins, occludin, and ZO-1, are beneficial to maintain the physical integrity of the intestinal epidermal barrier. Disruption or defects in the intestinal barrier integrity may cause microbial imbalances and other harmful substances to cross the epithelial barrier, leading to activation of immune cells and intestinal inflammation (Kabat et al., 2014; Suzuki, 2020). Khan *et al.* (Khan et al., 2021) reported that ammonia nitrogen exposure caused significant downregulation of *claudin* and *occludin* in *Mauremys sinensis*. Similarly, Ding *et al.* (Ding et al., 2021) found that ammonia nitrogen stress resulted in sparse and shortened intestinal villi, downregulation of tight junction genes, and increased cell permeability in *Trachemys scripta elegans*. In this study, compared with the control group, the intestinal villi of yellow catfish in the ammonia nitrogen exposure group were sparse and shorter, the cavities between the microvilli were obvious and the tight junctions were blurred. The gene expression of tight sealing proteins (*Occludin*, *Claudin-1* and *ZO-1*) was upregulated, while the gene expression of pore-forming protein (*Claudin-2*) was downregulated. These results indicate that ammonia nitrogen stress damages the integrity of intestinal mucosal physical barrier, enhances paracellular permeability, and may induce inflammation.

Inflammation is not only a universal defense response to stress, but also an indispensable part for tissue repair (Lv et al., 2017). External stimulation can induce cytokine maturation and participate in the regulation of inflammatory response. Numerous studies have shown that the expression level of cytokines can be considered as effective biomarkers of aquatic organisms’ inflammatory responses (Zhang et al., 2015; Jin et al., 2017). IL-1β, IL-8 and TNF-α are the major pro-inflammatory factors, which play significant roles in the development of inflammatory and autoimmune diseases (Weber et al., 2010). Previous study found that ammonia nitrogen stress caused apoptosis and increased expression of *TNF*, *IL-1β* and *IL-8* in *Pelteobagrus fulvidraco* (Li et al., 2020). This study likewise yielded similar results. With the increase of ammonia nitrogen level, the expression levels of *TNF-α*, *IL-1β* and *IL-8* increased, and the tissue sections exhibited obvious inflammatory cell infiltration. Furthermore, Zap-70 immunohistochemistry indicated that ammonia nitrogen stress induced recruitment of T/NK cells to the site of inflammation. Taken together, these findings demonstrate that ammonia nitrogen stress induces a strong inflammatory response in the intestine of yellow catfish.

An interesting finding in this study was that, the low concentration group (0.5 mg/L) had more severe damage to the intestinal physical barrier than the high concentration group (2.5 mg/L), including lower expression of tight junction related genes, sparser and shorter microvilli, and more blurred mucosal barrier. However, the high concentration group (2.5 mg/L) showed a trend of mucosal barrier repair. In fact, a growing number of studies have shown that certain inflammatory cytokines contribute to barrier protection. Although IL-17A is known to be an inflammatory cytokine, but it also protects the mucosal barrier by affecting *Occludin* expression (Lee et al., 2015). Shih *et al.* (Dudakov et al., 2015) revealed that following gastrointestinal infection, *IL-22* was upregulated, promoting tissue regeneration, barrier formation, and antibacterial defense. Jarry *et al.* (Jarry et al., 2008) revealed that IL-10 could provide barrier protection and participate in epithelial repair induced by intestinal inflammation. Therefore, highly regulated spatiotemporal interactions between mucosal cytokines (including pro-inflammatory and anti-inflammatory factors) may benefit epithelial barrier repair. In this study, the expression level of pro-inflammatory factors (*IL-1β, IL-8, TNF-α*) rose with the increase of ammonia nitrogen concentration, and the expression level of tight junction related genes (*Occludin*, *Claudin-1*, *ZO-1*) decreased first and then increased. Moreover, the high ammonia concentration group also had the highest expression of anti-inflammatory factor *IL-10*. Considering the above points, we believe that at the sampling time point, fish in the high concentration group were in the later stages of inflammation resolution and tissue repair period, whereas fish in the low concentration group were still in the major inflammatory stage.

Under natural conditions, TAN and pNH_3_ levels in intestinal chyme are very high (Bucking and Wood, 2012), exceeding the water ammonia levels considered toxic to fish (Randall and Tsui, 2002). However, the intestine can still maintain homeostasis and normal morphological structure, implying that the intestinal epithelium should be well adapted to deal with high ammonia levels at the enteric surface. Interestingly, our work suggests that ammonia nitrogen has a significant toxic effect on the intestine of yellow catfish. Whether this effect is direct or indirect, and whether it is mediated by blood ammonia or cortisol, remains unclear and needs further investigation.

## 5 Conclusion

In this study, we systematically evaluated the pathological changes after ammonia nitrogen challenge in yellow catfish after an ammonia nitrogen challenge at the histological, molecular, and ultrastructural levels, as well as the inflammation-related immune response. In conclusion, this investigation demonstrates that ammonia nitrogen stress can damage the intestinal mucosal barrier of yellow catfish and induce intestinal inflammation.

## Data Availability

The datasets presented in this study can be found in online repositories. The names of the repository/repositories and accession number(s) can be found in the article/Supplementary Material.

## References

[B1] Administration M. o. A. F. (2021). Statistics of China Fishery yearbook. Beijing: China Agriculture Press.

[B2] Andreozzi G. Antonucci R. Affatato C. Gargiulo G. Battaglini P. (1994). The effect of cadmium on the intestine of *Carassius auratus* . Anat. Histol. Embryol. 23 (2), 102–111. 10.1111/j.1439-0264.1994.tb00242.x 7978344

[B3] Barišić J. Filipović Marijić V. Mijošek T. Čož-Rakovac R. Dragun Z. Krasnići N. (2018). Evaluation of architectural and histopathological biomarkers in the intestine of brown trout (*Salmo trutta* Linnaeus, 1758) challenged with environmental pollution. Sci. Total Environ. 642, 656–664. 10.1016/j.scitotenv.2018.06.045 29909333

[B4] Bischoff S. C. Barbara G. Buurman W. Ockhuizen T. Schulzke J. D. Serino M. (2014). Intestinal permeability--a new target for disease prevention and therapy. BMC Gastroenterol. 14, 189. 10.1186/s12876-014-0189-7 25407511 PMC4253991

[B5] Bucking C. Wood C. M. (2012). Digestion of a single meal affects gene expression of ion and ammonia transporters and glutamine synthetase activity in the gastrointestinal tract of freshwater rainbow trout. J. Comp. Physiol. B 182 (3), 341–350. 10.1007/s00360-011-0622-y 21994022

[B6] Camilleri M. Madsen K. Spiller R. Greenwood-Van Meerveld B. Verne G. N. (2012). Intestinal barrier function in health and gastrointestinal disease. Neurogastroenterol. Motil. 24 (6), 503–512. 10.1111/j.1365-2982.2012.01921.x 22583600 PMC5595063

[B7] Chen J. Zang X. Gengdong H. U. Jianhong Q. U. Fan L. (2011). The immune response of GIFT *Oreochromis niloticus* and its susceptibility to Streptococcus iniae under stress in different ammonia. Ecol. Environ. Sci. 10.1016/S1671-2927(11)60313-1

[B8] Chen Q. L. Luo Z. Zheng J. L. Li X. D. Liu C. X. Zhao Y. H. (2012). Protective effects of calcium on copper toxicity in *Pelteobagrus fulvidraco*: copper accumulation, enzymatic activities, histology. Ecotoxicol. Environ. Saf. 76 (2), 126–134. 10.1016/j.ecoenv.2011.10.007 22019308

[B9] Chen Q. Zhao H. Huang Y. Cao J. Wang G. Sun Y. (2016). Effects of dietary arginine levels on growth performance, body composition, serum biochemical indices and resistance ability against ammonia-nitrogen stress in juvenile yellow catfish (*Pelteobagrus fulvidraco*). Anim. Nutr. 2 (3), 204–210. 10.1016/j.aninu.2016.07.001 29767042 PMC5941038

[B10] Ding L. Huang Z. Lu Y. Liang L. Li N. Xu Z. (2021). Toxic effects of ammonia on intestinal health and microbiota in red-eared slider (*Trachemys scripta* elegans). Chemosphere 280, 130630. 10.1016/j.chemosphere.2021.130630 33930609

[B11] Dudakov J. A. Hanash A. M. van den Brink M. R. (2015). Interleukin-22: immunobiology and pathology. Annu. Rev. Immunol. 33, 747–785. 10.1146/annurev-immunol-032414-112123 25706098 PMC4407497

[B12] Ghosh S. S. Wang J. Yannie P. J. Cooper R. C. Sandhu Y. K. Kakiyama G. (2021). Over-expression of intestinal alkaline phosphatase attenuates atherosclerosis. Circ. Res. 128 (11), 1646–1659. 10.1161/circresaha.120.317144 33834851

[B13] Gonçalves A. R. N. Marinsek G. P. de Souza Abessa D. M. de Britto Mari R. (2020). Adaptative responses of myenteric neurons of Sphoeroides testudineus to environmental pollution. Neurotoxicology 76, 84–92. 10.1016/j.neuro.2019.10.008 31669307

[B14] Hongxing G. Xiafei L. Jialing L. Zhenquan C. Luoyu G. Lei L. (2021). Effects of acute ammonia exposure on antioxidant and detoxification metabolism in clam Cyclina sinensis. Ecotoxicol. Environ. Saf. 211, 111895. 10.1016/j.ecoenv.2021.111895 33476851

[B15] Jarry A. Bossard C. Bou-Hanna C. Masson D. Espaze E. Denis M. G. (2008). Mucosal IL-10 and TGF-beta play crucial roles in preventing LPS-driven, IFN-gamma-mediated epithelial damage in human colon explants. J. Clin. Invest. 118 (3), 1132–1142. 10.1172/jci32140 18259614 PMC2230656

[B16] Jin Y. Wu S. Zeng Z. Fu Z. (2017). Effects of environmental pollutants on gut microbiota. Environ. Pollut. 222, 1–9. 10.1016/j.envpol.2016.11.045 28086130

[B17] Kabat A. M. Srinivasan N. Maloy K. J. (2014). Modulation of immune development and function by intestinal microbiota. Trends Immunol. 35 (11), 507–517. 10.1016/j.it.2014.07.010 25172617 PMC6485503

[B18] Kathyayani S. A. Poornima M. Sukumaran S. Nagavel A. Muralidhar M. (2019). Effect of ammonia stress on immune variables of Pacific white shrimp Penaeus vannamei under varying levels of pH and susceptibility to white spot syndrome virus. Ecotoxicol. Environ. Saf. 184, 109626. 10.1016/j.ecoenv.2019.109626 31536848

[B19] Khan I. Huang Z. Liang L. Li N. Ali Z. Ding L. (2021). Ammonia stress influences intestinal histomorphology, immune status and microbiota of Chinese striped-neck turtle (Mauremys sinensis). Ecotoxicol. Environ. Saf. 222, 112471. 10.1016/j.ecoenv.2021.112471 34229168

[B20] Kim J. H. Rhee J. S. Dahms H. U. Lee Y. M. Han K. N. Lee J. S. (2012). The yellow catfish, *Pelteobagrus fulvidraco* (siluriformes) metallothionein cDNA: molecular cloning and transcript expression level in response to exposure to the heavy metals Cd, Cu, and Zn. Fish. Physiol. Biochem. 38 (5), 1331–1342. 10.1007/s10695-012-9621-5 22367486

[B21] Kleinhenz L. S. Trenfield M. A. Mooney T. J. Humphrey C. L. van Dam R. A. Nugegoda D. (2018). Acute ammonia toxicity to the larvae (glochidia) of the tropical Australian freshwater mussel Velesunio spp. Using a modified toxicity test protocol. Environ. Toxicol. Chem. 37 (8), 2175–2187. 10.1002/etc.4175 29786863

[B22] Kołacińska K. Koncki R. (2014). A novel optoelectronic detector and improved flow analysis procedure for ammonia determination with Nessler's reagent. Anal. Sci. 30 (10), 1019–1022. 10.2116/analsci.30.1019 25312634

[B23] Kruatrachue M. Rangsayatorn N. Pokethitiyook P. Upatham E. S. Singhakaew S. (2003). Histopathological changes in the gastrointestinal tract of fish, Puntius gonionotus, fed on dietary cadmium. Bull. Environ. Contam. Toxicol. 71 (3), 561–569. 10.1007/s00128-003-8795-z 14567583

[B24] Lee J. S. Tato C. M. Joyce-Shaikh B. Gulen M. F. Cayatte C. Chen Y. (2015). Interleukin-23-Independent IL-17 production regulates intestinal epithelial permeability. Immunity 43 (4), 727–738. 10.1016/j.immuni.2015.09.003 26431948 PMC6044435

[B25] Lee P. T. Yamamoto F. Y. Low C. F. Loh J. Y. Chong C. M. (2021). Gut immune system and the implications of oral-administered immunoprophylaxis in finfish aquaculture. Front. Immunol. 12, 773193. 10.3389/fimmu.2021.773193 34975860 PMC8716388

[B26] Li M. Zhang M. Qian Y. Shi G. Wang R. (2020). Ammonia toxicity in the yellow catfish (*Pelteobagrus fulvidraco*): the mechanistic insight from physiological detoxification to poisoning. Fish. Shellfish Immunol. 102, 195–202. 10.1016/j.fsi.2020.04.042 32330626

[B27] Liu S. (2022). Effects of ammonia nitrogen stress on gill respiration and immune function of yellow catfish (*Pelteobagrus fulvidraco*) and its mechanism. Cheng du: Sichuan Agricultural University.

[B28] Livak K. J. Schmittgen T. D. (2001). Analysis of relative gene expression data using real-time quantitative PCR and the 2(-Delta Delta C(T)) Method. Methods 25 (4), 402–408. 10.1006/meth.2001.1262 11846609

[B29] Lu J. Yao T. Shi S. Ye L. (2022). Effects of acute ammonia nitrogen exposure on metabolic and immunological responses in the Hong Kong oyster Crassostrea hongkongensis. Ecotoxicol. Environ. Saf. 237, 113518. 10.1016/j.ecoenv.2022.113518 35447473

[B30] Lv Z. Wei Z. Zhang Z. Li C. Shao Y. Zhang W. (2017). Characterization of NLRP3-like gene from Apostichopus japonicus provides new evidence on inflammation response in invertebrates. Fish. Shellfish Immunol. 68, 114–123. 10.1016/j.fsi.2017.07.024 28705721

[B31] Omonijo F. A. Liu S. Hui Q. Zhang H. Lahaye L. Bodin J. C. (2019). Thymol improves barrier function and attenuates inflammatory responses in porcine intestinal epithelial cells during lipopolysaccharide (LPS)-Induced inflammation. J. Agric. Food Chem. 67 (2), 615–624. 10.1021/acs.jafc.8b05480 30567427

[B32] Peterson L. W. Artis D. (2014). Intestinal epithelial cells: regulators of barrier function and immune homeostasis. Nat. Rev. Immunol. 14 (3), 141–153. 10.1038/nri3608 24566914

[B33] Pustiglione Marinsek G. Moledo de Souza Abessa D. Gusso-Choueri P. K. Brasil Choueri R. Nascimento Gonçalves A. R. D'Angelo Barroso B. V. (2018). Enteric nervous system analyses: new biomarkers for environmental quality assessment. Mar. Pollut. Bull. 137, 711–722. 10.1016/j.marpolbul.2018.11.015 30503489

[B34] Qian L. Miao L. Abba B. S. A. Lin Y. Jiang W. Chen S. (2021). Molecular characterization and expression of sirtuin 2, sirtuin 3, and sirtuin 5 in the Wuchang bream (*Megalobrama amblycephala*) in response to acute temperature and ammonia nitrogen stress. Comp. Biochem. Physiol. B Biochem. Mol. Biol. 252, 110520. 10.1016/j.cbpb.2020.110520 33045325

[B35] Randall D. J. Tsui T. K. (2002). Ammonia toxicity in fish. Mar. Pollut. Bull. 45 (1-12), 17–23. 10.1016/s0025-326x(02)00227-8 12398363

[B36] ShenpingCao D. Zhao D. Huang R. Xiao Y. Xu W. Liu X. (2021). The influence of acute ammonia stress on intestinal oxidative stress, histology, digestive enzymatic activities and PepT1 activity of grass carp (Ctenopharyngodon idella). Aquac. Rep. 20, 100722. 10.1016/j.aqrep.2021.100722

[B37] Suzuki T. (2020). Regulation of the intestinal barrier by nutrients: the role of tight junctions. Anim. Sci. J. 91 (1), e13357. 10.1111/asj.13357 32219956 PMC7187240

[B38] Varghese F. Bukhari A. B. Malhotra R. De A. IHC Profiler (2014). IHC profiler: an open source plugin for the quantitative evaluation and automated scoring of immunohistochemistry images of human tissue samples. PLoS One 9 (5), e96801. 10.1371/journal.pone.0096801 24802416 PMC4011881

[B39] Wang S. Li X. Zhang M. Jiang H. Wang R. Qian Y. (2021). Ammonia stress disrupts intestinal microbial community and amino acid metabolism of juvenile yellow catfish (*Pelteobagrus fulvidraco*). Ecotoxicol. Environ. Saf. 227, 112932. 10.1016/j.ecoenv.2021.112932 34700169

[B40] Weber A. Wasiliew P. Kracht M. (2010). Interleukin-1beta (IL-1beta) processing pathway. Sci. Signal 3 (105), cm2. 10.1126/scisignal.3105cm2 20086236

[B41] Wester P. W. Canton J. H. (1991). The usefulness of histopathology in aquatic toxicity studies. Comp. Biochem. Physiol. C Comp. Pharmacol. Toxicol. 100 (1-2), 115–117. 10.1016/0742-8413(91)90135-g 1677840

[B42] Wood C. M. Liew H. J. De Boeck G. Hoogenboom J. L. Anderson W. G. (2019). Nitrogen handling in the elasmobranch gut: A role for microbial urease. J. Exp. Biol. 222, jeb194787. 10.1242/jeb.194787 30530835

[B43] Wu Y. Tang L. Wang B. Sun Q. Zhao P. Li W. (2019). The role of autophagy in maintaining intestinal mucosal barrier. J. Cell Physiol. 234 (11), 19406–19419. 10.1002/jcp.28722 31020664

[B44] Zhang S. Jin Y. Zeng Z. Liu Z. Fu Z. (2015). Subchronic exposure of mice to cadmium perturbs their hepatic energy metabolism and gut microbiome. Chem. Res. Toxicol. 28 (10), 2000–2009. 10.1021/acs.chemrestox.5b00237 26352046

[B45] Zhang S. Lü B. Chao G. Q. Chen F. M. Chen M. Y. Chen H. Q. (2011). The effects of milk and milk products on non-steroidal anti-inflammatory drug induced intestinal damage in rats. Zhonghua Nei Ke Za Zhi 50 (9), 771–775. 10.1038/cdd.2010.68 22176967

[B46] Zhang T. Yan Z. Zheng X. Wang S. Fan J. Liu Z. (2020). Effects of acute ammonia toxicity on oxidative stress, DNA damage and apoptosis in digestive gland and gill of Asian clam (Corbicula fluminea). Fish. Shellfish Immunol. 99, 514–525. 10.1016/j.fsi.2020.02.046 32092406

[B47] Zhang T. Zhang Y. Xu J. Yan Z. Sun Q. Huang Y. (2021). Toxic effects of ammonia on the intestine of the Asian clam (Corbicula fluminea). Environ. Pollut. 287, 117617. 10.1016/j.envpol.2021.117617 34174666

[B48] Zhong L. (2022). Effects of ammonia nitrogen stress on gill excretion and osmotic pressure regulation of yellow catfish (*Pelteobagrus fulvidraco*). Cheng du: Sichuan Agricultural University.

[B49] Zhou X. Zhang G. R. Ji W. Shi Z. C. Ma X. F. Luo Z. L. (2021). Expression and function analysis of interleukin-17a/F1, 2, and 3 genes in yellow catfish (*Pelteobagrus fulvidraco*): distinct bioactivity of recombinant IL-17a/F1, 2, and 3. Front. Immunol. 12, 626895. 10.3389/fimmu.2021.626895 34267744 PMC8276262

[B50] Zimmerli S. Bernet D. Burkhardt-Holm P. Schmidt-Posthaus H. Vonlanthen P. Wahli T. (2007). Assessment of fish health status in four Swiss rivers showing a decline of brown trout catches. Aquat. Sci. 69 (1), 11–25. 10.1007/s00027-006-0844-3

