# Supplementary material for: Ammonia nitrogen stress damages the intestinal mucosal barrier of yellow catfish (Pelteobagrus fulvidraco) and induces intestinal inflammation
Source: Front Physiol. 2023 Sep 19;14:1279051. doi: 10.3389/fphys.2023.1279051 (PMC10542119; doi:10.3389/fphys.2023.1279051)
Supplement: Supplementary file 1 [file Table1.DOCX]

Supplementary Materials

**SI MATERIALS AND METHODS**

**Nessler reagent**. Dissolved 16.0g sodium hydroxide (NaOH) in 50 mL water and cooled to room temperature. Dissolved 7.0 g potassium iodide (KI) and 10.0 g mercury iodide (HgI2) in water, stirred and slowly added to the above 50 mL NaOH solution, diluted to 100 mL with water.

**Potassium sodium tartrate solution** (ρ = 500 g/L). Accurately weighed 50.0g potassium sodium tartrate (KNaC4H6O6•4H2O), dissolved in 100 mL water, boiled to remove ammonia, cooled sufficiently and diluted to 100 mL.

**Nessler reagent colorimetry method.** Prepared eight 50 mL colorimetric tubes, added 0.00 mL, 0.50 mL, 1.00 mL, 2.00 mL, 4.00 mL, 6.00 mL, 8.00 mL and 10.00 mL of ammonia nitrogen standard solution (10 µg/mL), respectively. Then added distilled water to 50 mL mark, and the ammonia nitrogen concentration in the corresponding colorimetric tubes were 0 mg/L, 0.1 mg/L, 0.2 mg/L, 0.4 mg/L, 0.8 mg/L, 1.2 mg/L, 1.6 mg/L and 2 mg/L, respectively. Added 1.0 mL potassium sodium tartrate solution and 1.0 mL Nessler reagent and mixed thoroughly. After 10 min, the absorbance of each tube (420 nm) was tested using distilled water as a reference. The standard curve was drawn with the corrected absorbance as the X-axis and the corresponding ammonia nitrogen concentration (mg/L) as the Y-axis. 50 mL of water was taken from each plastic bucket for absorbance test and the actual concentration of ammonia nitrogen was calculated according to the standard curve.

**SUPPLEMENTARY Tables**

**Table S1** The daily detected concentration of ammonia nitrogen

|  | TAN concentration (mg/L) | | | | | |
| --- | --- | --- | --- | --- | --- | --- |
| Date | 2.5mg/L group | | | 0.5mg/L group | | |
| Day1 | 2.545 | 2.578 | 2.523 | 0.647 | 0.562 | 0.524 |
| Day2 | 2.506 | 2.588 | 2.566 | 0.785 | 0.431 | 0.530 |
| Day3 | 2.505 | 2.598 | 2.647 | 0.526 | 0.505 | 0.495 |
| Day4 | 2.409 | 2.525 | 2.576 | 0.746 | 0.503 | 0.480 |
| Day5 | 2.559 | 2.537 | 2.547 | 0.510 | 0.556 | 0.562 |
| Day6 | 2.710 | 2.572 | 2.769 | 0.562 | 0.641 | 0.660 |
| Day7 | 2.248 | 2.429 | 2.435 | 0.562 | 0.412 | 0.733 |
| Day8 | 2.544 | 2.514 | 2.527 | 0.562 | 0.530 | 0.628 |
| Day9 | 2.519 | 2.545 | 2.565 | 0.674 | 0.628 | 0.779 |
| Day10 | 2.489 | 2.475 | 2.553 | 0.654 | 0.595 | 0.477 |
| Day11 | 2.665 | 2.577 | 2.655 | 0.569 | 0.589 | 0.556 |
| Day12 | 2.400 | 2.442 | 2.507 | 0.400 | 0.477 | 0.400 |
| Day13 | 2.488 | 2.553 | 2.625 | 0.366 | 0.386 | 0.477 |
| Day14 | 2.357 | 2.612 | 2.488 | 0.549 | 0.464 | 0.517 |
| Day15 | 2.448 | 2.514 | 2.422 | 0.543 | 0.510 | 0.418 |
| Day16 | 2.272 | 2.586 | 2.448 | 0.562 | 0.438 | 0.523 |
| Day17 | 2.547 | 2.599 | 2.618 | 0.582 | 0.490 | 0.667 |
| Day18 | 2.527 | 2.553 | 2.684 | 0.621 | 0.687 | 0.602 |
| Day19 | 2.913 | 2.540 | 2.717 | 0.582 | 0.562 | 0.536 |
| Day20 | 2.953 | 2.730 | 2.723 | 0.589 | 0.779 | 0.589 |
| Day21 | 2.632 | 2.730 | 2.736 | 0.569 | 0.510 | 0.562 |
| Day22 | 2.802 | 2.743 | 2.678 | 0.654 | 0.530 | 0.641 |
| Day23 | 2.723 | 2.573 | 2.651 | 0.888 | 0.582 | 0.667 |
| Day24 | 2.658 | 2.796 | 2.740 | 0.601 | 0.726 | 0.621 |
| Day25 | 2.730 | 2.625 | 2.799 | 0.615 | 0.641 | 0.648 |
| Day26 | 2.608 | 2.678 | 2.672 | 0.723 | 0.610 | 0.792 |
| Day27 | 2.601 | 2.568 | 2.677 | 0.554 | 0.630 | 0.640 |
| Day28 | 2.473 | 2.467 | 2.499 | 0.439 | 0.501 | 0.543 |

**Table S2** Primers used for gene expression analysis by qRT-PCR in intestinal samples of yellow catfish

| Gene | Name | Forward Sequence | Reverse Sequence | | Efficiency |
| --- | --- | --- | --- | --- | --- |
| *IL-8*  *KY218792.1* | Interleukin 8 | CACCACGATGAAGGCTGCAACTC | | TGTCCTTGGTTTCCTTCTGG | 96% |
| *IL-10*  *KY218793.1* | Interleukin 10 | CTCCTCCCCCTGAGGATTCA | | CGGATCACGGCGTATGAAGA | 95% |
| *IL-1β*  *JQ730738.1* | Interleukin 1β | TAGGCATAGAGGAGGTAA | | AAGGTGTTCAGGGAGTCA | 95% |
| *TNF-α*  *XM_027165847.2* | Tumor necrosis factor*-α* | ATAACCCACGCCTATGACTG | | GGCTATGACTCGCAACACTT | 94% |
| *ZO-1*  *XM_047810112.1* | Zonula occludens 1 | GCGAACTCTCTGAACAGCCT | | TGTGTGTGTGCAGGAGGTTT | 96% |
| *Occludin*  *XM_027141818.2* | Occludin | CGAGCGAGAGACTACGACAC | | TCCAGGAATTGTGGGCTTCC | 95% |
| *Claudin-2*  *XM_047804898.1* | Claudin domain containing 2 | TCAGGACGACAGACGAGGA | | AGGCACACCCACAGGAACT | 96% |
| *Claudin-1*  *XM_047815958.1* | Claudin domain containing 1 | ACGCTAACAACGGCTCAGA | | CCTTACATTCAGACACCACCTT | 94% |
| *β-actin*  *XM_027148463.2* | β-actin | TTCGCTGGAGATGATGCT | | CGTGCTCAATGGGGTACT | 97% |

**SUPPLEMENTARY FIGURE**

**
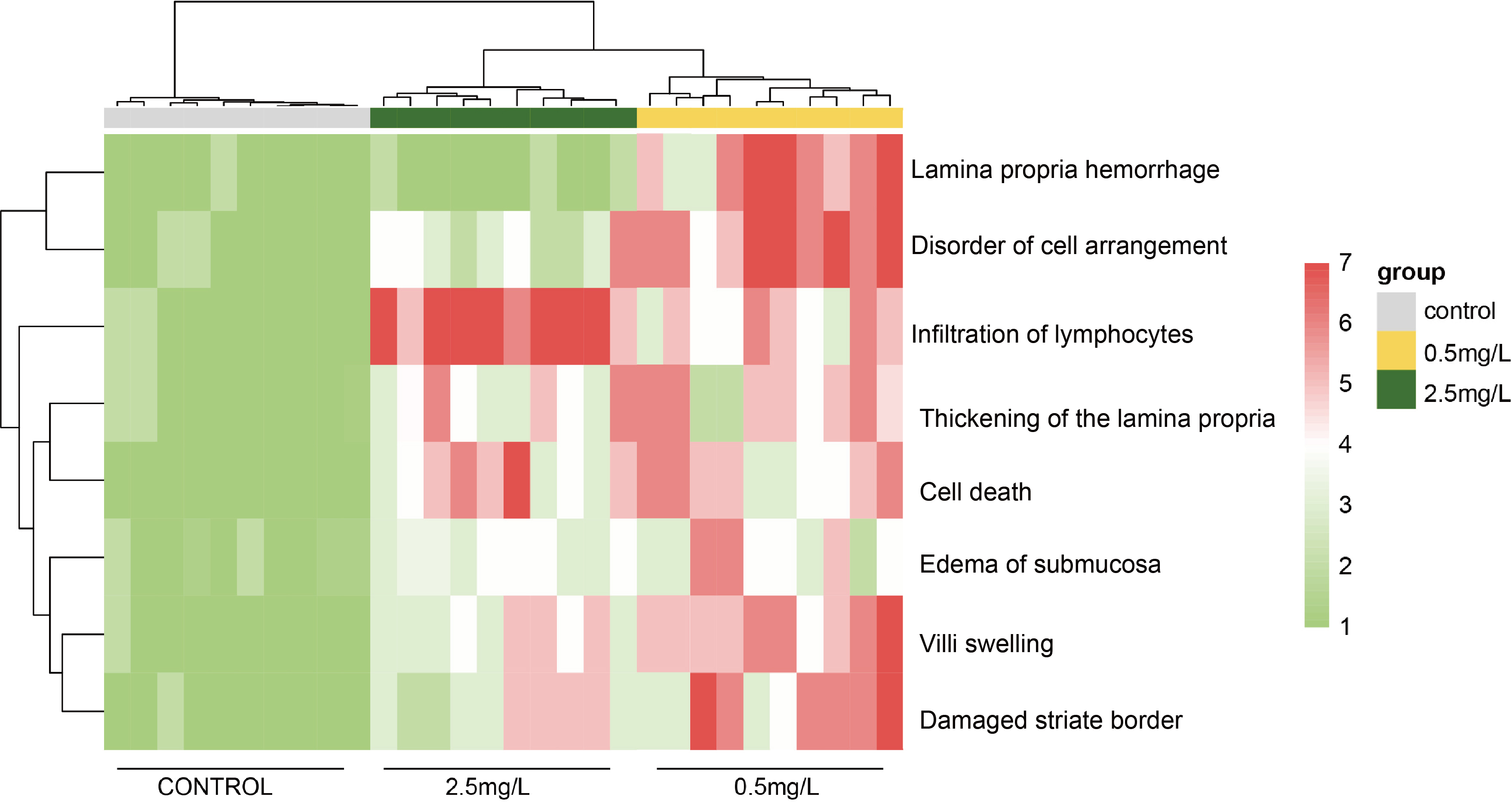
**

**Fig. S1.** Heat map of pathological changes, including lamina propria hemorrhage, disorder of cell arrangement, infiltration of lymphocytes, thickening of the lamina propria, cell death, edema of submucosa, villi swelling, and damaged striate border.

**
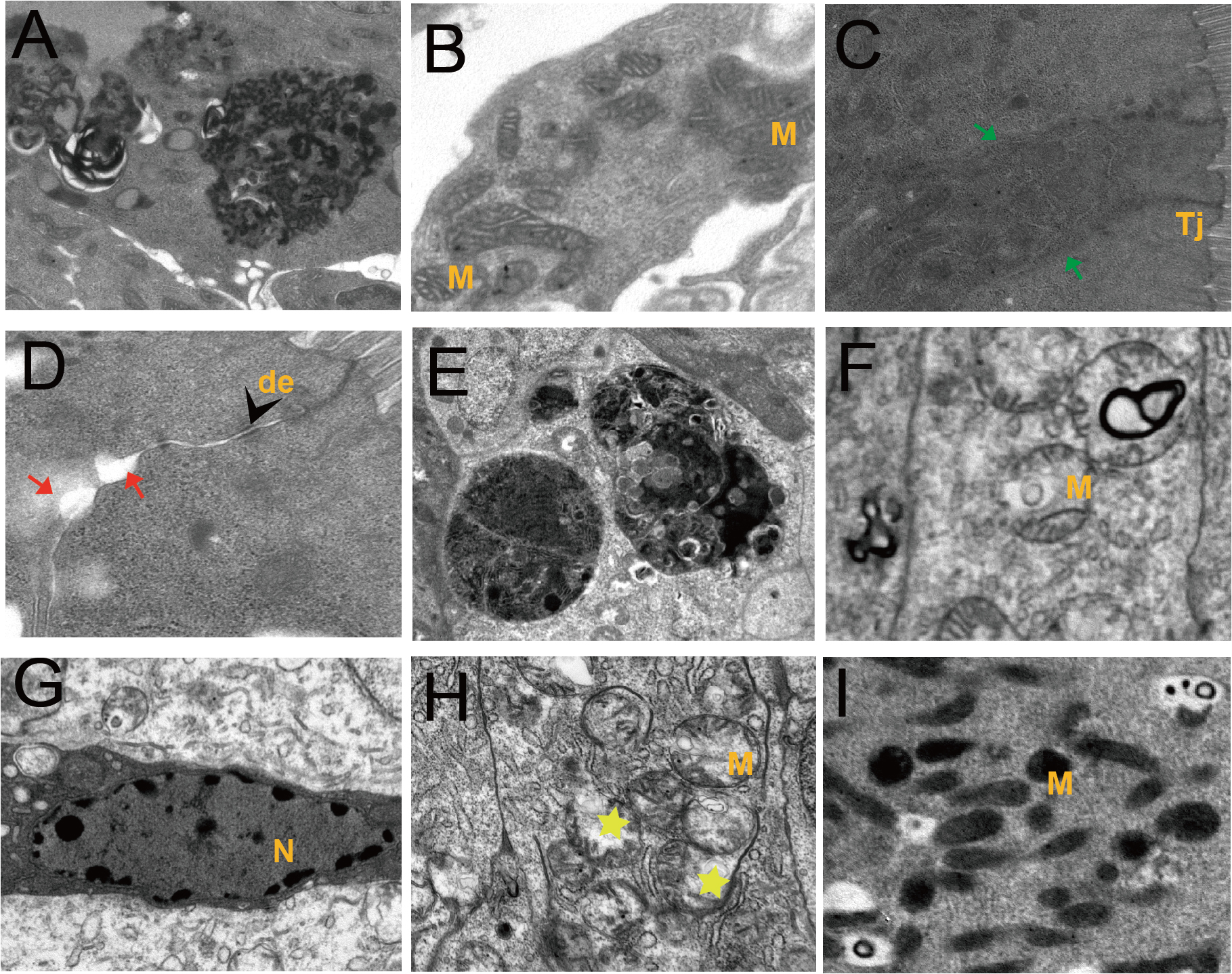
**

**Fig. S2.** Ultrapathological changes of intestine after ammonia nitrogen exposure

(A_-_D) The ultrastructural pathological changes of intestine in 0.5 mg/L group: (A), Necrosis; (B), Mitochondrial cristae contraction; (C), Tight junctions fuzzy (green arrow); (D), Tight junctions gap (red arrow). (E-I) The ultrastructural pathological changes of intestine in 2.5 mg/L group: (E), Necrosis; (F), Autophagy; (G), Apoptosis; (H), Mitochondrial myelinoid lesions (yellow star); (I), Mitochondrial necrosis.

N, nucleus; M, mitochondria; Tj, tight junctions; de, desmosomes.
